# Draft Genome Sequence of Bifidobacterium longum ZJ1, Isolated from a Centenarian in Anhui, China

**DOI:** 10.1128/MRA.00878-19

**Published:** 2019-08-15

**Authors:** Zeyu Jin, Wei Li, Wei Wang, Baolin Sun

**Affiliations:** a Chorain Health Technology Co., Ltd., Hefei, Anhui, China; b School of Life Sciences, University of Science and Technology of China, Hefei, Anhui, China; University of Rochester School of Medicine and Dentistry

## Abstract

Here, we present the complete genome sequence of a Bifidobacterium longum isolate, that of strain ZJ1, and this strain showed a cholesterol degradation ability that is greater than that of five strains we chose for comparison (Bifidobacterium longum 536, B. infantis 1912, B. longum 1941, B. breve ATCC 15698, B. infantis ATCC 17930). The draft genome of strain ZJ1 consists of 2,414,672 bp, with 2,042 protein-coding genes, 69 noncoding RNA genes, and 60.16% G+C content.

## ANNOUNCEMENT

Bifidobacterium spp. have been shown to improve human health conditions and provide protection against infection and potentially health-promoting metabolites (1). The *Bifidobacterium* genus contains probiotics which could improve the clinical symptoms in patients with mild to moderate active ulcerative colitis (2). Bifidobacterium longum is one of the most abundant species of the Bifidobacterium genus (3).

Bifidobacterium longum ZJ1 was isolated from a fecal sample from a 105-year-old male from Hefei, Anhui, China. The isolate was cultivated on de Man, Rogosa, and Sharpe (MRS) agar (Oxoid, Basingstoke, United Kingdom) supplemented with 0.05% (wt/vol) l-cysteine under aerobic conditions for 48 h at 35°C. Genomic DNA of the cultured isolate was extracted using the Wizard genomic DNA purification kit (Promega, San Luis Obispo, CA, USA), according to the manufacturer’s instructions.

The genome sequence of Bifidobacterium longum ZJ1 was sequenced using a PacBio RS II platform (20-kb SMRTbell template) and an Illumina HiSeq 4000 platform (TruSeq DNA PCR-free 350-bp library) at the Beijing Genomics Institute (BGI, Shenzhen, China). Genomic DNA was sheared with a g-TUBE (Covaris) and purified using AMPure PB magnetic beads (Beckman Coulter) to construct a 20-kb library. The libraries were further size selected utilizing BluePippin (Sage Scientific, Beverly, MA), with a cutoff size of 10 kb, and sequenced on a PacBio RS II sequencer. Also, the DNA sample was randomly fragmented and ligated with 5′ and 3′ adapters to construct the sequencing library for Illumina HiSeq 4000 platform paired-end sequencing.

A total of 8,396,464 reads were obtained from Illumina and low-quality reads (bases with quality lower than 20, >40%; N content, >10%) were filtered using SOAPnuke v1.5.2 (4) (default settings). In total, 128,072 subreads were obtained from PacBio sequencing, and the subreads with less than 1 kb were removed. The *N*_50_ and *N*_90_ values of the subreads are 1,885 bp and 3,551 bp, respectively. The package proovread v2.12 (5) (-t 4 –coverage 60 –mode sr) in the program pbdagcon was used for self-correction. Draft genomic unitigs were assembled using the Celera Assembler v8.3 (6) (doTrim_initialQualityBased = 1, doTrim_finalEvidenceBased = 1, doRemoveSpurReads = 1, doRemoveChimericReads = 1, d properties -U), and then GATK v1.6-13 (7) (-cluster 2 -window 5 -stand_call_conf 50 -stand_emit_conf 10.0 -dcov 200 MQ0> = 4) was used for single-base corrections to improve the accuracy of the genome sequences.

The assembled genome consists of one circular chromosome of 2,414,672 bp, with a G+C content of 60.16%. There are 2,042 protein-coding genes, as predicted by Glimmer3 v3.02 (8), within the genome of Bifidobacterium longum ZJ1. In total, 12 rRNA genes, 56 tRNA genes, and 1 small RNA (sRNA) gene were recognized by tRNAscan-SE v1.3.1 (9) [–Spec_tag(BAOG) –o *. tRNA –f * .tRNA.structure], RNAmmer v1.2 (10) (–s Species –m Type –gff *. rRNA.gff –f *.rRNA.fq), and the Rfam database v9.1 (11) (–p blastn –W 7 –e 1 –v 10000 –b 10000 –m 8 –i subfile –o *.blast.m8).

Two incomplete prophages were found using the PHAge Search Tool (PHAST) v2013.03.20 (12) (default settings); one prophage has 12,784 bp, and the other prophage has 30,265 bp. CRISPRFinder v0.4 (13) (default settings) was used to identify 7 CRISPR sequences. One has 30 spacers, one has 7 spacers, and the others have only 1 spacer.

The synteny of B. longum ZJ1 and 4 other B. longum strains (B. longum 105-A, B. longum AH1206, B. longum BORI, and B. longum KACC91563) was determined using MUMmer v3.22 (14) (-b 200 -c 65 –extend -l 20), and BLAST core/pan genes of these 5 strains were clustered using CD-HIT v4.6.6 (15) (-c 0.5 -n 3 -p 1 -g 1 -d 0 -s 0.7 -aL 0.7 -aS 0.7) with a threshold of 50% pairwise identity and 0.7 length difference cutoff in amino acids. Finally, 1,356 core genes were identified.

### Data availability.

This whole-genome project has been deposited at DDBJ/EMBL/GenBank under accession number CP040235. The BioProject accession number is PRJNA539831. Raw sequence reads are deposited at DDBJ/EMBL/GenBank under accession numbers SRR9051180 and SRR9051181.
